# Matrix Metalloproteinase-1 Polymorphism (-1607G) and Disease Severity in Non-Cystic Fibrosis Bronchiectasis in Taiwan

**DOI:** 10.1371/journal.pone.0066265

**Published:** 2013-06-11

**Authors:** Meng-Heng Hsieh, Pai-Chien Chou, Chun-Liang Chou, Shu-Chuan Ho, Wen-Ching Joa, Li-Fei Chen, Te-Fang Sheng, Horng-Chyuan Lin, Tsai-Yu Wang, Po-Jui Chang, Chun-Hua Wang, Han-Pin Kuo

**Affiliations:** 1 Department of Thoracic Medicine, Chang Gung Medical Foundation, Taipei, Taiwan; 2 School of Respiratory Therapy, College of Medicine, Taipei Medical University, Taipei, Taiwan; University of Giessen Lung Center, Germany

## Abstract

**Objectives:**

Bronchiectasis is characterized by an irreversible dilatation of bronchi and is associated with lung fibrosis. *MMP-1* polymorphism may alter its transcriptional activity, and differentially modulate bronchial destruction and lung fibrosis.

**Design:**

To investigate the association of *MMP-1* polymorphisms with disease severity in non-cystic fibrosis (CF) bronchiectasis patients, 51 normal subjects and 113 patients with bronchiectasis were studied. The associations between *MMP-1* polymorphisms, lung function, and disease severity evaluated by high resolution computed tomography (HRCT) were analyzed.

**Results:**

The frequency of *MMP-1*(-1607G) allele was significantly higher in patients with bronchiectasis than normal subjects (70.8% vs 45.1%, p<0.01). Forced expiratory volume in 1 second (FEV1) was decreased in bronchiectasis patients with 1G/1G (1.2±0.1 L, n = 14) and 1G/2G (1.3±0.1 L, n = 66) genotypes compared to the 2G/2G genotype (1.7±0.1 L, n = 33, p<0.01). Six minute walking distance was decreased in bronchiectasis patients with 1G/1G and 1G/2G compared to that of 2G/2G genotype. Disease severity evaluated by HRCT score significantly increased in bronchiectasis patients with 1G/1G and 1G/2G genotypes compared to that of 2G/2G genotype. Bronchiectasis patients with at least one *MMP-1* (-1607G) allele showed increased tendency for hospitalization. Serum levels of pro-MMP-1, active MMP-1 and TGF-β1 were significantly increased in patients with bronchiectasis with 1G/1G and 1G/2G genotype compared with 2G/2G genotype or normal subjects. Under IL-1β stimulation, peripheral blood monocytes from subjects with 1G/2G or 1G/1G genotype secreted higher levels of TGF-β1compared to subjects with 2G/2G genotype.

**Conclusion:**

This is the first report to address the influence of *MMP-1* polymorphisms on lung function and airway destruction in non-CF bronchiectasis patients. Bronchiectasis patients with *MMP-1*(-1607G) polymorphism may be more vulnerable to permanent lung fibrosis or airway destruction due to the enhanced MMP-1 and TGF-β1 activity. Upregulated MMP-1 activity results in proteolytic destruction of matrix, and leads to subsequent fibrosis.

## Introduction

Bronchiectasis is a chronic inflammatory lung disease characterized by irreversible dilatation of the bronchi and, in most cases, by persistent production of purulent sputum [1], and associated with chronic cough, hemoptysis, breathlessness, and tiredness in patients [2]. Due to inflammation induced by bacterial infections, various proteinases are synthesized and released by activated neutrophils and resident cells, such as fibroblasts [3]. Additionally, severe bronchiectasis has been associated with the presence of low-molecular weight gelatinases reflecting *in vivo* metalloproteinase activation and/or the presence of microbial-derived gelatinolytic proteinases [4]. The host defense reaction leads to an uncontrolled degradation of components of the lung extracellular matrix (ECM), which leads to bronchiectasis [5]. Therefore, bronchiectasis is often associated with tissue remodeling, and increased turnover of the ECM, however the precise mechanisms are currently poorly understood.

Matrix metalloproteinases (MMPs) comprise of more than 20 human zinc-dependent proteolytic enzymes that are associated with degradation of the ECM, contributing to the development of tissue remodeling and lung fibrosis [6], [7]. MMPs have been shown to regulate leukocyte migration to sites of infection, but in excessive amounts may contribute to tissue destruction and fibrosis [8], [9], [10]. A number of MMPs, such as MMP-1 (interstitial collagenase), MMP-9 (gelatinase B) and MMP-12 (macrophage metalloelastase), are secreted by monocytes and macrophages [9], and further upregulated by cytokines, TNF-α and IL-1β [11], [12], which degrade fibrillar collagens [9], [10], [13]. The interstitial collagenases are therefore key initiators of ECM degradation, and comprises of the fibroblast-type collagenase/MMP-1 derived from fibroblasts [14], [15], and monocyte/macrophages [9], [10]. Collagenase (MMP-1) and neutrophil-type collagenase/MMP-8 are found in broncholaveolar lavage fluid from patients with bronchiectasis [16]. Furthermore, airway epithelium adjacent to TB granuloma also expresses high levels of MMP-1 [17], contributing to the rupture of pulmonary cavity formation and tissue destruction [18], [19]. Overexpression of MMP-1 and MMP-12 has been associated with pulmonary emphysema and airway remodeling [20], [21]. MMPs are therefore implicated in a range of pulmonary diseases, in relation to alterations in alveolar structure or abnormal healing response or lung fibrosis [10], [22], [23]. Thus, changes in the level or activities of these MMPs may play a critical role in the altered collagen metabolism of pulmonary fibrosis and airway remodeling in patients with bronchiectasis.

Polymorphisms of the promoters of the *MMP-1* genes might have allele-specific effects on the regulation of MMP gene transcription, and are associated with proteolytic destruction of chronic obstructive pulmonary disease [21], with clinical characteristics of sarcoidosis and tuberculosis [24], and with the development and progression of cancers [25]. A 1G/2G polymorphic site has been found to be located in a core recognition sequence of the binding sites for transcription factors that controls the level of MMP-1 expression [26]. Our recent work has shown that patients with endobronchial TB granuloma containing 1G genotype of *MMP-1* polymorphism had a greater risk of developing tracheobronchial stenosis through up-regulation of MMP-1 activity [18]. Patients with *MMP-1*(-1607G) polymorphism are more vulnerable to more extensive lung fibrosis 1 year after anti-tuberculosis treatment, which may be related to increased MMP-1 activity, leading to enhanced destruction of the matrix with subsequent fibrosis [10]. During the process of lung fibrosis, local overexpression of cytokines and/or growth factors stimulates resident pulmonary fibroblast to synthesize an increased amount of ECM. Transforming growth factor (TGF)-β1 is a key mediator responsible for the ECM changes seen in lung fibrosis [27]. There is altered production of soluble proteins such as TGF-β1, MMPs, and a tissue inhibitor of MMPs (TIMP-1), as well as deposition of fixed proteins, such as fibronectin and tenascin in nthe airway remodeling process of asthma [28]. Excessive secretion of MMP-1 may also increase the fibroblast or structural cells of lung thereby increasing TGF-β1 secretion [29]. The fibrotic process of lung may partly result from the interaction between MMP-1 and TGF-β1. The links between the airways remodeling, lung fibrosis and *MMP-1* polymorphisms have been recognized in several studies, including ours. However, little is known whether these reported polymorphisms are associated with the severity of bronchiectasis.

Therefore, the aim of the study was to investigate the association of *MMP-1* polymorphisms with disease severity in patients with non-cystic fibrosis bronchiectasis. We hypothesized that *MMP-1* polymorphisms might lead to increased expression of MMP genes, and through increased activity of the enzymes, that would contribute to the lung fibrosis and/or airway destruction in patients with bronchiectasis.

## Methods

### Study Population

113 patients (aged 55.0±1.6 years, 69 women and 44 men) with bronchiectasis diagnosed by high-resolution computed tomography (HRCT), were recruited from our outpatient clinic of Chang Gung Memorial Hospital. Inclusion criteria included daily sputum production>10 ml; and "steady-state" of bronchiectasis (<10% alteration of 24 h sputum volume, FEV1, and FVC, and in the absence of deterioration in respiratory symptoms at baseline visits). The exclusion criteria were unreliable clinic attendance, other unstable systemic diseases, Young's syndrome (bronchiectasis with sinusitis, and obstructive azoospermia), evidence of other organ system disease, including chronic dysfunction of the pancreas or liver or intestine, or an electrolyte imbalance, disease onset before adolescence and family history, that would suggest cystic fibrosis (CF), regular user of inhaled corticosteroids, and known asthma defined according to American Thoracic Society guidelines. For analysis of lung function, patients with previous lung resection were excluded.

Fifty-one healthy volunteers (aged 52.3±1.9 years, 29 female and 22 male) from Taiwanese populations were enrolled. None of them had a history of chronic lung disease based on physical and chest radiographic examination. None of the participants was taking any antibiotics or chronic medication at the time of evaluation.

The study protocol was approved by the Institutional Ethics Committee of Chang Gung Memorial Hospital. Written informed consent was signed and obtained from all participants.

### Clinical Assessment

Patients with bronchiectasis were followed in outpatient clinic on a monthly basis for 1 year. Six-minute walking test, including forced expiratory volume in one second (FEV1) and forced vital capacity (FVC), oxygen saturation before and after walking, was measured on entering the study. Patients withacute exacerbation in which hospitalization indicated was checked and recorded.

### High Resolution Computed Tomography (HRCT)

**Figure 1:**
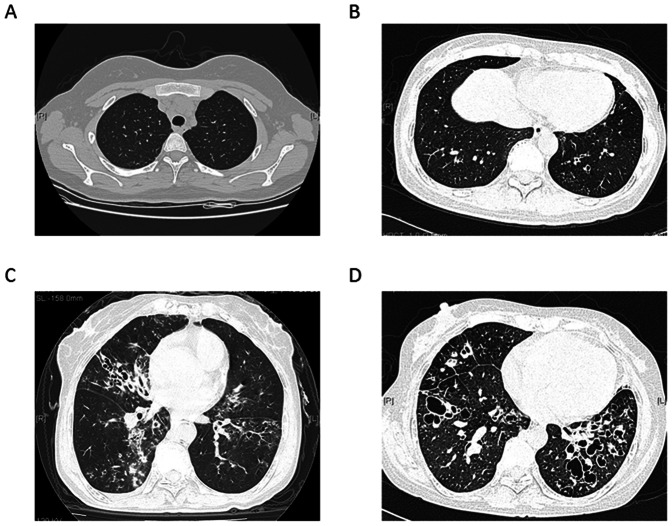
HRCT grading system for patients with bronchiectasis. Each lobe of both lungs is graded for bronchiectatic changes on a scale of 0 to 3 (lingula was scored as a separate lobe), giving a maximum of 18 points. (A) Represents score 0: no bronchiectasis. (B) Represents score 1 in one lobe: one bronchopulmonary segment involved (C) Represents score 2 in one lobe: more than one bronchopulmonary segment involved. Total Score was 8 in this figure due to four lobes involved. (D) Represents score 3 in one lobe: grossly cystic bronchiectasis. Total Score was 12 in this figure due to three lobes involved.

A recent HRCT (within a week from entering the study) scan (GE 9800 Highlight Advanced; General Electric Medical Systems; Milwaukee, WI) in patients with bronchiectasis were assessed and scored by two radiologists who were blinded to all patient details concerning clinical and functional conditions. During the 7-day observation, none of our patients had an exacerbation of disease. Each lobe of both lungs (RUL, RML, RLL, LUL, Left lingual lobe, and LLL) is graded for bronchiectatic changes on a scale of 0 to 3 (0 = no bronchiectasis, 1 = one bronchopulmonary segment involved, 2 = more than one bronchopulmonary segment involved, and 3 = gross cystic bronchiectasis) (lingula was scored as a separate lobe), giving a maximum of 18 points [30] (Figure 1). Patients with emphysematous changes unrelated to bronchiectasis were excluded.

### Preparation and Culture of Peripheral Blood Monocytes (PBMs)

Heparinized blood (30 ml) was collected from normal subjects with 2G/2G (n = 8) or 1G/2G (n = 9) or 1G/1G (n = 9) genotypes of the *MMP-1* polymorphism. PBM cells were isolated on a Ficoll-Hypaque density gradient (Sigma, St Louis, MO, USA). The monocytes were purified by adherence to >90% purity.

### DNA Extraction and *MMP-1* (G-1607GG) Genotyping

DNA was extracted from PBMs using a standard phenol/chloroform protocol. The DNA sequence flanking in the polymorphic region of each gene was amplified by PCR. Negative controls without DNA template were included with each set of reactions. All analyses were performed in a blind fashion, with the investigator unawere of the origin of the specimens. Genotypes were confirmed by a second person not directly involved in the study, and by dideoxynucleotide sequencing for a representative number of samples of genotype. The primer used to amplify the *MMP-1* DNA (*G-1607 GG*) sequence single nucleotide polymorphisms (sense and anti-sense) was: (5′-TGCCACTTAGATGACCAAATTG-3′ and 5′-GATTCCTGT- TTTCTTTCTGCGT-3′) [10], [18]. Amplification was performed in a 20 µl volume containing 100–200 ng of genomic DNA, 7.5 µM of each primer, 2.5 µM dNTPs, 10×polymerase chain reaction (PCR) buffer and 1 U of *Taq* polymerase. The solution was incubated for 1.5 min at 94°C, followed by 30 PCR cycles (0.5 min each at 94°C), 30 s at 56°C and 30 s at 72°C, with a final extension for 5 min at 72°C. PCR products were electrophoresized on a non-denaturing polyacrylamide gel stained with DNA Silver Staining Kit (Amersham Biosciences AB, Sweden). Method validity was checked by sequence analysis, using an Applied Biosystems 3730 series DNA Analyzers (Applied Biosystems, Foster City, CA).

### Measurement of MMP-1 and TGF-β1 Levels by ELISA

Pro- or active-MMP-1 and TGF-β1 levels in serum or culture supernatant were measured by ELISA (R&D Systems, Minneapolis, USA) according to the manufacturer’s instructions. The lower level of sensitivity is <10 pg/ml for MMP-1 and <10 pg/ml for TGF-β1. To study TGF-β1 released from PBMs, monocytes (5×10^5^ cells/ml) were incubated with IL-1β (0, 2, 10 and 50 ng/ml, R&D Systems, Minneopolis, USA) for 48 hrs. Supernatants were collected, and TGF-β1 levels were measured in duplicate wells in 12-well plastic tissue culture plates and cultured in an incubator at 5% CO_2_ and 37°C for 48 hrs.

### Statistical Analysis

Data were expressed as the mean±SE. One-way analysis of variance (ANOVA) for mixed design was used to compare values of more than two different experimental groups. If variance among groups was noted, a Bonferroni test was used to determine significant differences between specific points within groups. The data were analyzed by Student’s t-test for paired or unpaired data. For data with uneven variation, a Mann-Whitney U-test or Wilcoxon signed rank test was used for unpaired or paired data, respectively. A P-value of less than 0.05 was considered statistically significant.

## Results

### Characteristics of Participants

The gender, age and smoking status of those enrolled in the study were similar among normal and bronchiectatic populations (Table 1). Allele frequencies of *MMP-1*(-1607G) genotype between the bronchiectasis patients and controls are also shown (Table 1). The most common genotype of *MMP-1* polymorphism was 1G/2G in bronchiectasis patients (58.4%), and 2G/2G (54.9%) in the controls. The frequency of G allele was higher in bronchiectasis patients compared to that of normal subjects. The overall distribution of homozygotes and heterozygotes for each polymorphism was consistent with Hardy-Weinberg equilibrium.

**Table 1:** Characteristics of normal subjects and bronchiectasis patients.

	Normal subjects (N = 51)	Bronchiectasis (N = 113)	P value
Gender, Female (%)	29 (56.9)	69 (61.1)	0.728
Age, Mean±SE	52.3±1.9	55.0±1.6	0.090
Smoking status (%)	17 (33.3)	36 (31.9)	0.859
*MMP-1* genotype2G/2G (%)1G/2G (%)1G/1G (%)G allele (%)	28 (54.9)17 (33.3)6 (11.7)23 (45.1)	33 (29.2)66 (58.4)14 (12.4)80 (70.8)	0.005

### Association of *MMP-1* (-1067GG) Polymorphisms and Number of Involved Lobe in Bronchiectasis

The extent of bronchiectasis is shown in Table 2. 32 patients had only one lobe involved, while the remaining 81 patients had more than one lobe involved. There was a higher association for having either one or two copies of *MMP-1*(-1607G) polymorphisms in bronchiectasis patients with more than one lobe involved compared to those with only one lobe involved (81.5% *vs.* 43.8%, p = 0.0003) (Table 2).

**Table 2:** Association of *MMP-1* (-1067GG) polymorphisms and number of involved lobe in bronchiectasis.

Number of lobe involved	Genotype of *MMP-1* polymorphisms			
	2G/2G	1G/2G	1G/1G	P value
**One lobe, (N = 32), N (%)**	18 (56.2)	11 (34.4)	3 (9.4)	0.0003
**More than one lobe, (N = 81), N (%)**	15 (18.5)	55 (67.9)	11 (13.6)	

### *MMP-1* (-1607G) Polymorphisms and Lung Function, Walking Distance, CT Score and Hospitalization

The gender, age, duration of disease and smoking habit distribution were similar among the three groups based on the genotype of *MMP-1*(-1607G) polymorphisms (Table 3). FVC (1G/2G type, 1.7±0.1 L; 1G/1G type, 1.7±0.1 L, p<0.0001), and FEV1 (1G/2G type, 1.3±0.1 L; 1G/1G type, 1.2±0.1 L, p = 0.005) was significantly lower in bronchiectasis patients with at least one -1607G of *MMP-1* polymorphism compared to those with 2G/2G genotype (FVC, 2.3±0.1 L; FEV1, 1.7±0.1 L) (Table 3). The 6-minute walk distance and pre-exercise oxygen saturation were significantly decreased in bronchiectasis patients having at least one G allele. Disease extent on HRCT measured by CT score showed higher disease severity in the patients with 1G/2G and 1G/1G genotype of *MMP-1* (-1607G) polymorphism. Over the follow-up of one year, patients having at least one -1607G of *MMP-1* showed increased risk of hospitalization compared to patients with 2G/2G genotype (Table 3).

**Table 3:** Association of *MMP-1* (-1607G) polymorphisms with lung function, walking distance, CT score and hospitalization.

	2G/2G (N = 33)		1G/2G (N = 66)	1G/1G (N = 14)	P value
Gender, male (%)	15 (45.4)	22 (33.3)		7 (50.0)	0.336
Age, years	56.4±2.1	55.0±1.6		59.9±3.0	0.418
Smoking status (%)	13 (39.4)	17 (25.8)		6 (42.9)	0.250
Duration, years	12.5±0.7	13.1±0.6		13.3±1.8	0.827
FVC, L	2.3±0.1	1.7±0.1		1.7±0.1	0.0007
FVC, % pred.	79.4±3.0	62.0±2.5		55.7±3.2	<0.0001
FEV1, L	1.7±0.1	1.3±0.1		1.2±0.1	0.005
FEV1, % pred.	73.1±3.8	55.6±3.0		49.0±3.4	0.0004
FEV1/FVC, %	74.4±2.1	72.5±1.7		70.3±3.9	0.603
Saturation, %Pre-exercisePost-exercise	96.4±0.387.6±1.3	94.0±0.784.3±1.4		92.7±1.884.1±2.7	0.0470.289
Walking distance (Meter)	513.3±12.6	453.4±11.2		400.7±33.5	0.0003
CT scoring	3.4±0.7	9.4±0.6		11.3±0.7	<0.0001
Hospitalization (times/year/person)	0.05±0.03	0.5±0.1		0.6±0.2	0.0067
Mortality, person	0	4		1	0.335

Abbreviations: FVC: forced vital capacity; L: liter; % pred.: percentage of predicted value; FEV1: forced expiratory volume in the first second; CT: computed tomography.

### Pro-MMP-1, and Active MMP-1 Serum Levels

**Figure 2:**
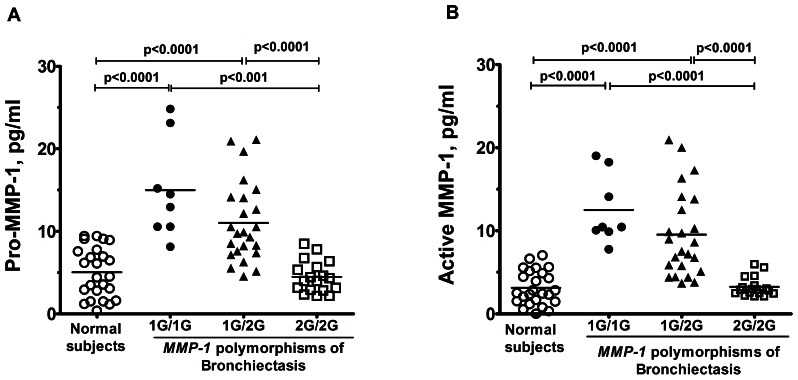
The serum level of pro-MMP-1 and active MMP-1 in normal subjects and patients with bronchiectasis. (A) The serum level of pro-MMP-1 measured in normal participants and in bronchiectasis patient with 1G/1G genotype, 1G/2G genotype, and 2G/2G genotype alternatively. (B) The serum level of active MMP-1 measured in normal participants, and in bronchiectasis patient with 1G/1G genotype, 1G/2G genotype, and 2G/2G genotype alternatively. Individualized p-values were marked on difference over different clinical settings.

Serum levels of pro-MMP-1 and active MMP-1 were significantly increased in patients with bronchiectasis with 1G/1G genotype (15.0±2.1 pg/ml; 12.5±1.5 pg/ml, respectively, n = 8, p<0.0001) and 1G/2G genotype (11.0±1.0 pg/ml; 9.5±1.1 pg/ml, respectively, n = 24, p<0.0001), compared to those with 2G/2G genotype (4.5±0.5 pg/ml; 3.2±0.3 pg/ml, n = 18) or normal subjects (5.0±0.6 pg/ml; 3.1±0.4 pg/ml, n = 25) (Figure 2A and B).

### TGF-β1 Serum Levels

**Figure 3:**
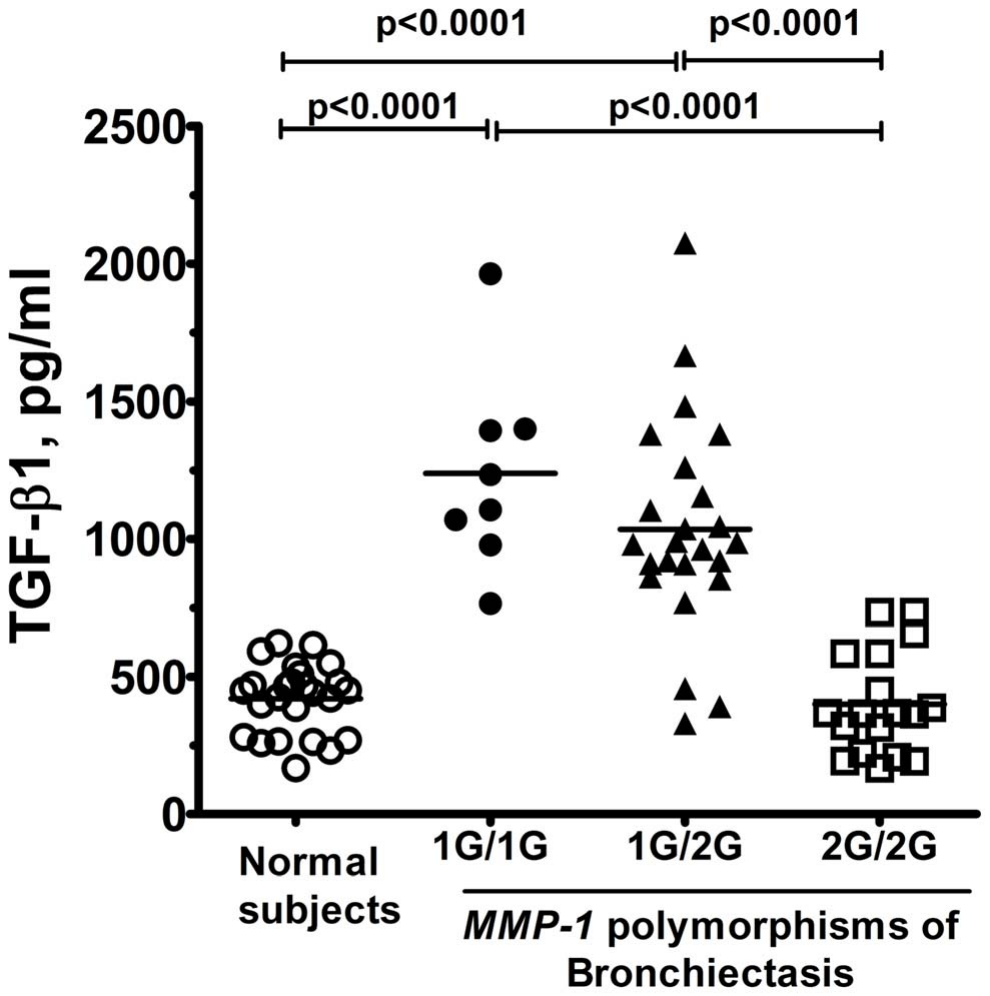
The serum level of TGF-β1 in normal subjects and patients with bronchiectasis. The serum level of TGF-β1 in patients with bronchiectasis having at least one -1607G of *MMP-1* was measured and compared to different clinical setting. Individual difference was marked as p-values among different settings.

There were increased levels of TGF-β1 in bronchiectasis patients with 1G/1G genotype (1239.0±361.5 pg/ml, n = 8, p<0.0001 to normal subjects and 2G/2G genotype), and 1G/2G genotype (1035.0±386.7 pg/ml, n = 24, p<0.0001 compared to normal subjects and 2G/2G genotype), compared to patients with 2G/2G genotype (400.3±185.5 pg/ml, n = 18) and normal subjects (419.8±126.0 pg/ml, n = 25) (Figure 3). The results demonstrated that bronchiectasis patients who have at least one -1607G of MMP-1 have higher TGF-β1 levels than those with bronchiectasis with 2G/2G genotype or normal subjects.

### Secreted TGF-β1 from IL-1β Stimulated Monocytes

**Figure 4:**
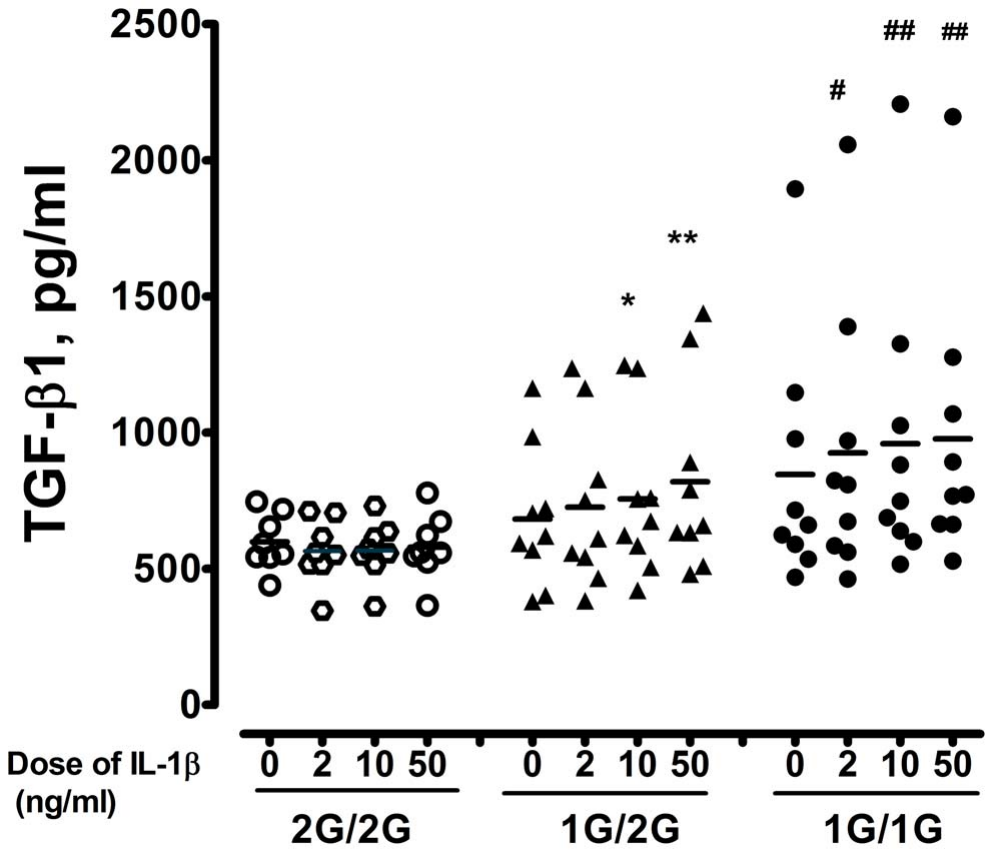
TGF-β1 levels secreted from monocytes under IL-1β stimulation for 48 hrs in normal subjects. PBMs were isolated from normal subjects, and stimulated with IL-1**β** (0, 2, 10 and 50 ng/ml alternatively) for 48 hours. Supernatants were collected and TGF-**β**1 level were measured. * denotes p<0.05, and ** denotes p<0.01 when compared to control in the 1G/2G genotype; and # denotes p<0.05 and ## denotes p<0.01 when compared to control in the 1G/1G genotype.

To investigate the differential effects on IL-1β inducible TGF-β1 levels on PBMs obtained from normal subjects with *MMP-1* polymorphisms, PBMs were stimulated for 48 hrs with various concentrations of IL-1β, and supernatants were collected for analysis. In participants with 2G/2G genotype, incubation of monocytes with various concentrations of IL-1β did not affect amounts of released TGF-β1. However, in normal subjects with 1G/2G genotype, 10 ng/ml and 50 ng/ml. IL-1β stimulation significantly upregulated TGF-β1 production compared to those without IL-1β stimulation (10 ng/ml IL-1β: 756.4±296.0 pg/ml, n = 9, p<0.05; 50 ng/ml IL-1β: 819.8±348.3 pg/ml, n = 9, p<0.05; without IL-1β: 682.3±254.8 pg/ml, n = 9) (Figure 4). Monocytes isolated from normal subjects with 1G/2G or 1G/1G genotype stimulated by IL-1β (10 and 50 ng/ml) released significantly higher TGF-β1 than those from subjects with the 2G/2G genotype (Figure 4).

## Discussion

We have demonstrated that the frequency of *MMP-1*(-1607G) polymorphism was associated with greater extent of disease and more lung destruction in patients with bronchiectasis. Bronchiectasis patients with the 1G genotype of *MMP-1* polymorphism were more vulnerable to subsequent advanced lung fibrosis or destruction, as well as frequency of hospital admission due to disease exacerbation. The serum levels of MMP-1 and TGF-β1 were higher in bronchiectasis patients with 1G/2G and 1G/1G genotype of *MMP-1* polymorphism. The finding that PBMs isolated from patients with 1G allele secreted higher levels of TGF-β1 after IL-1β stimulation compared to the 2G/2G genotype further confirmed the clinical importance of this polymorphism through excessive release of TGF-β1. This is the first report supporting this polymorphism of the promoter region of *MMP-1* as linked to the severity of bronchiectasis, and as being an independent risk factor for increased tissue destruction and lung fibrosis in bronchiectasis.

The frequency of 1G allele of MMP-1 polymorphism in our bronchiectasis patients, especially those with multilobar involvement (Table 2), was significantly different from that of healthy controls, indicating that the polymorphism of MMP-1 promoter is associated with the risk for developing bronchiectasis. Previously, Stankovic et al also reported that *MMP-1* polymorphism was associated with the risk of bronchiectasis [31]. The frequency of -1607GG allele was significantly higher in the group of patients than in the control subjects. By contrast, our results demonstrated that bronchiectasis patients with one or two copies of G allele of *MMP-1* polymorphism tended to have a greater extent of lung destruction, a rapid decline of pulmonary function and reduced exercise tolerance. The functional importance of this gene polymorphism is likely attributable to the enhanced proteolytic destruction of matrix, and leading to pulmonary and airway destruction, as well as subsequent fibrosis. MMP-1 degrades collagens I and III present in the lung parenchyma [32]. Up-regulation of *MMP-1 *gene and protein expression has been shown in human lung fibrosis [33], [34]. *MMP-1* polymorphism is associated with endobronchial TB that develops tracheobronchial stenosis [18], and with an increased risk for the development of lung fibrosis after TB infection [10]. These lines of evidence suggest that *MMP-1* polymorphism induced upregulation of MMP-1 activity is associated with post-inflammatory lung destruction and fibrogenesis.

The polymorphism at the promoter region of *MMP-1* (at position -1607) has been previously shown to alter gene expression [21]. The 2G allele results in greater transcriptional activity than the 1G allele, because the guanine insertion creates a binding site for the Ets family of transcription factors [26], [35]. Although several studies have indicated that the 2G polymorphism contributes to a rapid decline in lung function in cigarette smokers and in aggressive cancers [36], [37], [38], a recent study showed an increased prevalence among sarcoidosis patients with 1G/1G or 1G/2G genotype presenting with ocular or multi-organ involvement, while increased trend for cavity formation was also seen in TB patients with 1G/1G genotype [24]. Another report also suggested involvement of the *MMP-1*(-1607) 1G/2G polymorphism on the risk for developing oral cancer in the 1G allele European carriers [39]. Our previous report [10] demonstrated that PBMs from subjects with 1G/2G or 1G/1G genotype stimulated by IL-1β secreted higher levels of MMP-1, while MMP-1 release was not stimulated by IL-1β in subjects with 2G/2G genotype. Mostly important, the serum level of MMP-1 in our bronchiectasis patients with at least one G allele of *MMP-1* polymorphism was upregulated compared to that of 2G/2G genotype (Figure 2B). A plausible explanation for such an association is not obvious at the moment, but it may be related to the fact that in the absence of 2G allele, the expression of MMP-1 can be compensated by using alternative pathways and cis-acting sequences to achieve high levels of MMP-1 expression, contributing to a greater degradation of matrix. Indeed, high levels of MMP-1 expression in melanoma cells have been seen in cells homozygous for the 1G allele, which are mediated through both ERK1/2 and p38 mitogen-activated protein kinase pathways, while only the ERK pathway targets the 2G allele [40].

During the development of lung fibrosis, overexpression of cytokines and/or growth factors locally stimulates resident pulmonary fibroblasts to synthesize increased amounts of ECM. The homeostasis of lung fibrosis is tightly controlled by proteolytic degradation of existing ECM by MMPs, and inhibition of MMP activity by specific antiproteases of tissue inhibitors of metalloproteinase (TIMPs) [41], [42]. TGF-β1 may be a key mediator responsible for the ECM changes seen in lung fibrosis [41]. Our results found that the serum level of TGF-β1 from bronchiectasis patients with 1G/2G and 1G/1G genotype was higher than those of normal control or bronchiectasis patients with 2G/2G genotype (Figure 3). In asthma, altered production of soluble proteins such as TGF-β1, MMPs and TIMP-1, as well as deposition of fixed proteins, such as fibronectin and tenascin, has been demonstrated to be associated with subepithelial fibrosis [28]. Excessive secretion of MMP-1 may also lead to the fibroblasts or other structural cells of the lung to secrete TGF-β1 thereby further upregulating type I collagen and TIMP-1, and counter-regulating *MMP-1* gene transcription [43], [44]. In addition, PBMs from subjects with 1G/2G or 1G/1G genotype stimulated by IL-1β secreted higher levels of TGF-β1 (Figure 4), while MMP-1 release was not stimulated by IL-1β in subjects with 2G/2G genotype. These results support the possibility that the fibrotic process or airway destruction in bronchiectasis may partly result from the balance of interactions between MMP-1 and TGF-β1.

Although the number of patient is relatively small for a genetic polymorphism study, our results demonstrate that bronchiectasis patients with *MMP-1*(-1607G) polymorphism are more likely to develop a greater extent of lung fibrosis or airway destruction, which may be attributable to the production of MMP-1 and TGF-β1 activity and leading to proteolytic destruction of matrix. Further studies with a larger cohort of patients may be warranted to elucidate the possible involvement of MMP-1 in the pathogenesis of bronchiectasis. This polymorphism may be used as a genetic marker to evaluate of the severity of bronchiectasis. Genetic analysis may aid in the design of individualized forms of therapy and predict outcomes in the future.
